# Supplementary material for: Neural criticality from effective latent variables
Source: ArXiv. 2023 Oct 13:arXiv:2301.00759v3. Originally published 2023 Jan 2. Preprint. [Version 3] (PMC9882570)
Supplement: 1 [file NIHPP2301.00759V3-supplement-1.pdf]

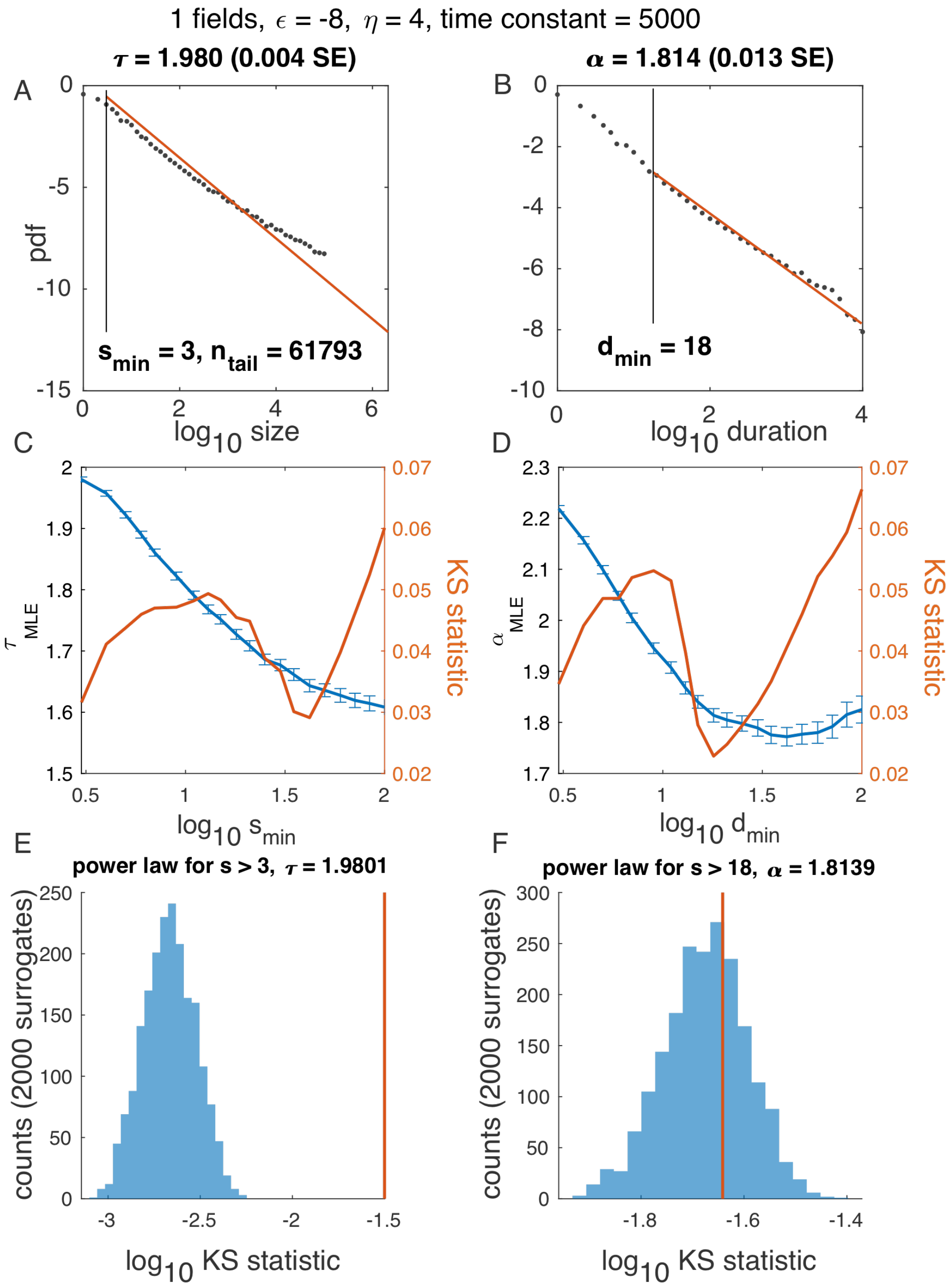

Figure 2–Figure supplement 1. Illustration of algorithm for determining τ and α, using one variable example in Fig. 2. **A-B:** Probability density function for avalanche size (A) and duration (B) on a log-log scale, with the best power law fit (red). **C-D:** In blue: Maximum likelihood exponent of a power-law model as a function of the minimum (lower cutoff) size (C) and duration (D). In red: KS statistics (see *Methods*) for each fit. “Best fit” is the power law with the minimum KS statistic. **E-F:** Surrogate data procedure. To generate each surrogate, samples were drawn from a power law with size / duration cutoff indicated (E, $S_{min} = 3$; F, $D_{min} = 18$) and the KS statistic was computed. Histograms illustrate KS statistic across surrogates (blue), while values derived from data are in red. Because the red line does not fall within the blue histogram, the hypothesis that the data is fitted well by a power law fit was rejected in E. At the same time, since the red line falls within the blue histogram in F, the hypothesis was accepted.

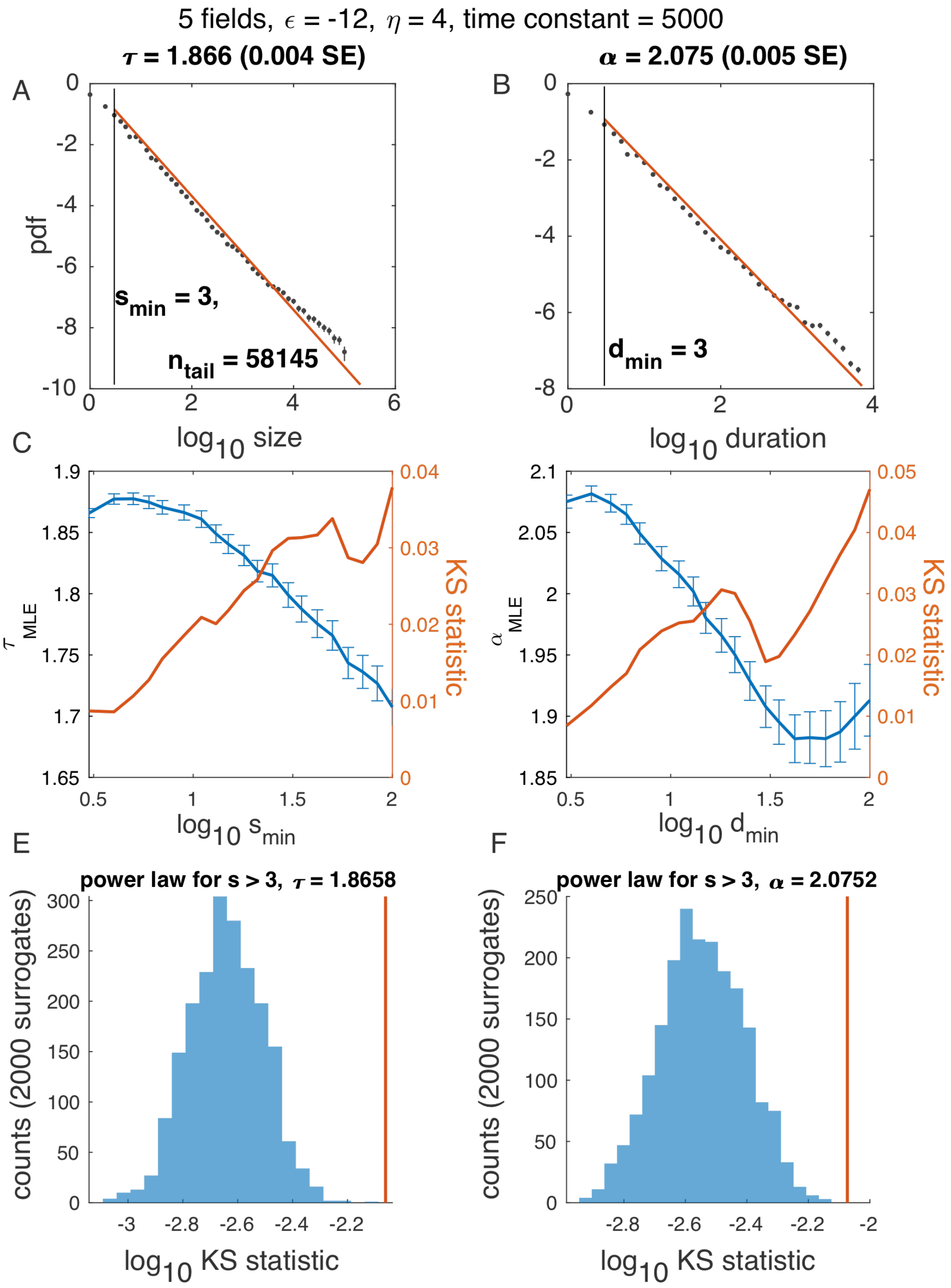

Figure 2–Figure supplement 2. Illustration of algorithm for determining τ and α, using example in Fig. 2, five latent variables. Notation the same as in Fig. 2-Fig. Supplement 3.

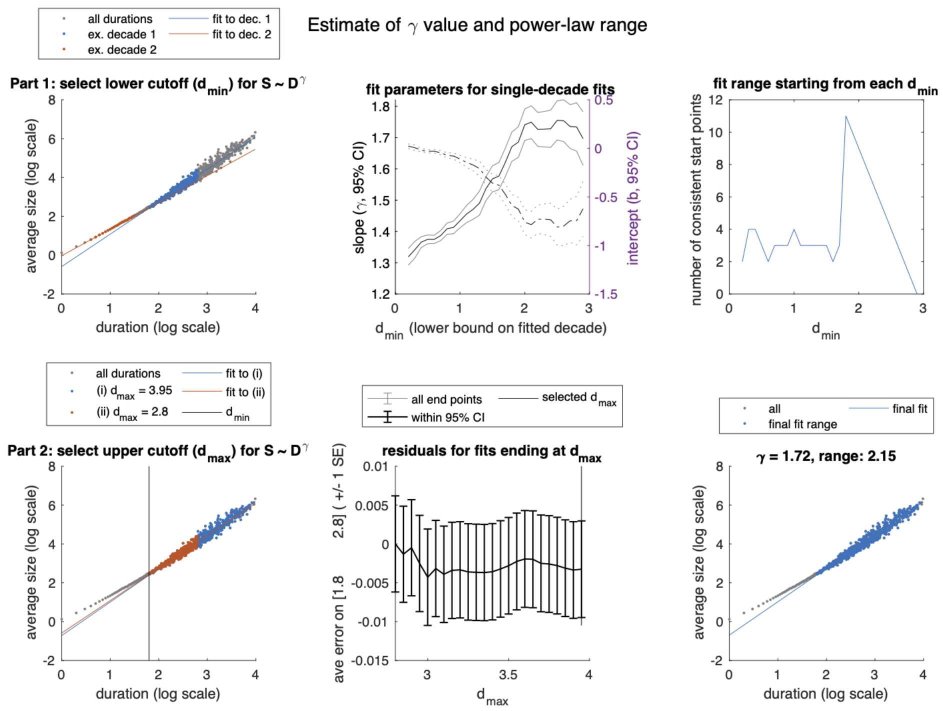

Figure 2–Figure supplement 3. Illustration of algorithm for fitting the exponent γ and determining the range, over which power law scaling of average size with duration is observed, using example in Fig. 2(A-D). **A-C:** Determining the lower bound, the minimum duration D_{min}. **A:** The relation $\log S = b + \gamma \log D$ was fit using linear least-squares, restricted to (overlapping) 1-decade ranges (blue, red: example decades). **B:** Confidence intervals for fit parameters (γ, b for fits starting at each value of D_{min}. **C:** Best value of $D_{\rm min}$ was selected based on how many subsequent start points yielded consistent slope/intercept values. **D-F:** Determining the upper bound, maximum duration D_{max}. **D:** Keeping D_{min} fixed based of value obtained in C, we test values of D_{max} up to the maximum duration event, and fit over the range $[D_{\rm min}, D_{\rm max}]$. **E:** Average residual over the fit range $[D_{\rm min}, D_{\rm min} + 1]$, calculated for each fit and plotted against the value of $D_{\rm max}$ used for that fit. The largest value of $D_{\rm max}$ without evidence of bias in the residual was then selected. **F:** Final fit and range.

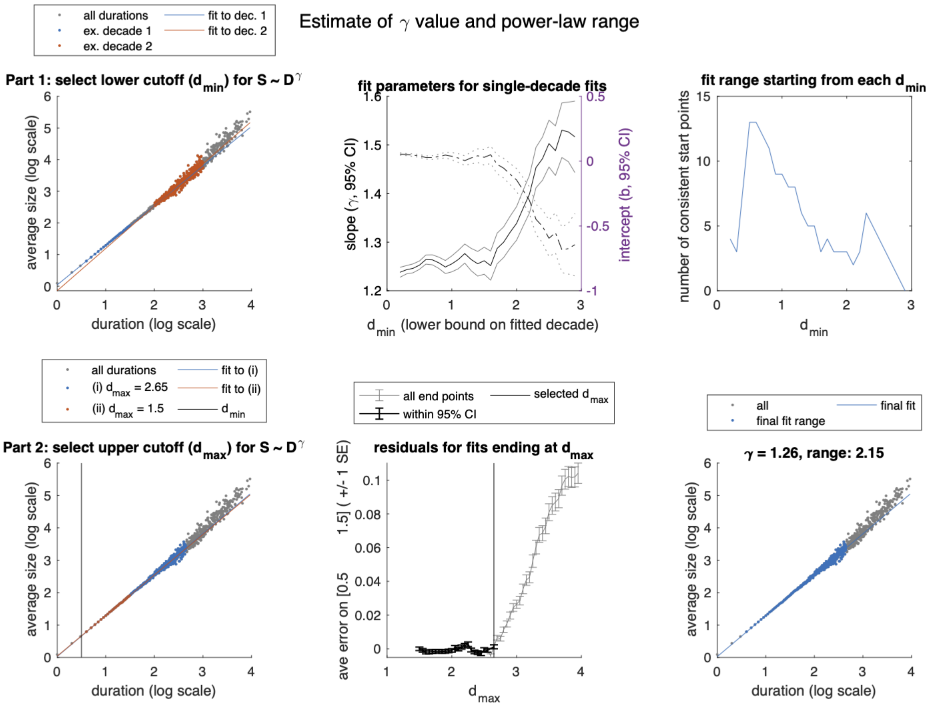

Figure 2–Figure supplement 4. Illustration of algorithm for fitting the exponent γ and determining the range, over which power-law scaling of average size with duration is observed, using example in Fig. 2 E-H. See Fig. 2-Fig. Supplement 3 for caption. In this example, a lower value of $D_{\min}$ was selected. Panel E, which was flat for Fig. 2-Fig. Supplement 3, now shows how extending the range to high values of $D_{\max}$ can generate systematic errors at the low range of the fit, even while having a high overall goodness of fit metric.

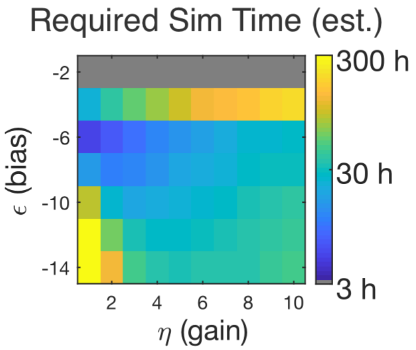

Figure 4–Figure supplement 1. Estimate of how long it takes to observe avalanche criticality at each combination of η and ϵ. We took a parameter combination with a low rate of avalanches but good apparent scaling ($\eta = 4$ and $\epsilon = -14$) and assumed that this is a reasonable estimate of the minimum number of observations (approximately 10^6 avalanches) required to observe scaling. To translate to observation length (in hours), we divided the number of avalanches observed in each full-length simulation by this minimum count and converted to a time using a time bin of 10 ms. Simulations were for a recorded population of 128 neurons. For this size of population, $\epsilon_0 = 5.2$.
